# Antimicrobial stewardship in long term care facilities: evidence based interventions, implementation tools, and impact metrics

**DOI:** 10.1017/ash.2025.10255

**Published:** 2025-12-22

**Authors:** Tristan T. Timbrook, Chelsea Campbell, Pamela Bailey

**Affiliations:** 1 Department of Pharmacy, Barnes-Jewish Hospital, Saint Louis, MO, USA; 2 Department of Pharmacotherapy, University of Utah College of Pharmacy, Salt Lake City, UT, USA; 3 South Carolina Department of Public Heath, Columbia, SC, USA; 4 Department of Internal Medicine, Prisma Health Midlands, Division of Infectious Diseases, Columbia, SC, USA; 5 University of South Carolina School of Medicine, Columbia, SC, USA

## Abstract

While Centers for Medicare and Medicaid Services-mandated Antimicrobial Stewardship Programs are now in place across U.S. skilled nursing facilities, reported high rates of compliance may mask persistent gaps in clinical effectiveness. This review summarizes evidence-based antimicrobial stewardship interventions, their impact, and practical tools to support implementation. Resources from Centers for Disease Control and Prevention, Agency for Healthcare Research and Quality, other frameworks, and proposed metrics are highlighted to guide effective stewardship in resource-variable nursing home settings.

## Introduction

Even though U.S. regulations under 42 CFR §483.80 Centers for Medicare and Medicaid Services (CMS) mandate structured Antimicrobial Stewardship Programs (ASPs) in skilled nursing facilities (SNFs), meeting regulatory minimums does not guarantee clinically effective stewardship.^1^ SNFs continue to exhibit substantial gaps in prescribing quality with approximately 1 out of every 10 residents are actively receiving an antibiotic and around 50% of prescriptions deemed inappropriate in indication, dose, or duration.^2,3^ Inappropriate antibiotic practices directly contribute to elevated rates of antimicrobial resistance, *Clostridioides difficile* infection increases, and worse patient outcomes.^4,5^

Following the 2016 CMS regulation self-reported progress has been substantial with adherence to stewardship best practices rising from 42% to 83% among facilities participating in the National Healthcare Safety Network (NHSN).^6,7^ Yet important gaps remain. A state-based survey beyond just NHSN facilities indicated only 67% of facilities report access to infectious diseases expertise, suggesting NHSN-reporting facilities may not be representative.^8^ Moreover, even among NHSN-reporting facilities less than one-third use electronic health records for antibiotic use tracking.^7^ These are not minor oversights as prospective audit with feedback from infectious diseases experts is a core driver of ASP success, and accurate tracking is essential for measuring impact.^9^ Consequently, program effectiveness varies widely despite CMS requirements.

While CMS regulations specifically target SNFs, the evidence base for effective antimicrobial stewardship spans the broader landscape of long-term care. Accordingly, this review synthesizes these evidence-based interventions for application across all long-term care facilities (LTCFs), grounding them in the specific CMS requirements for SNFs and highlighting practical, LTCF-specific tools for implementation and measurement.^10^

## Evidence on ASP interventions in LTCFs

The literature on antimicrobial stewardship in long-term care has expanded dramatically since the 2016 CMS regulations, with publications indexed by the National Library of Medicine rising from just ten in the three years prior to a peak of 63 in 2021.^11^ Recent systematic review literature (SLR) summaries as well as newer randomized controlled trials and public health government-sponsored quality improvement projects provide critical insights into the effectiveness of various interventions (Table 1). Across SLRs, most studies were conducted in the United States, ranging from 40% to 71% of studies.^12–17^ The number of studies included among SLRs ranged from 5 to 16 studies and 132 to 270 LTCFs reflected among the SLRs.

**Table 1.** Systematic literature reviews and meta-analyses of ASP interventions in LTCFs

Study	Design	Geographic location of included studies	No. studies/NHs*	Patient population	Interventions	Outcomes
**Feldstein 2017** ^10^	SLR	71% US	14/171	Majority UTI, ASB	ID consults, nursing communication modalities, education, clinical pathways	Reduction in Rx, increased in guideline adherence No change in mortality, *Clostridioides difficile* infection, or hospitalization
**Wu 2018** ^11^	SLR/MA	55% US	11/182	Majority UTI, followed by any infection, and NHAP	General ASP strategies	14% reduction in overall prescribing
**Nguyen 2019** ^12^	SLR	40% US	5/132	UTI, any infection, and NHAP	Education, guidelines, or prescribing report cards	Modest impact on antimicrobial guideline adherence and prevalence of prescribing Likely improvement in DDDs/1,000 resident-days No effect on mortality or hospitalization
**Crespo-Rivas** ^13^ ** 2021	SLR/MA	58% US	12/270	Majority UTI followed by all infections and respiratory	General ASP strategies	Change of −.47 prescriptions per 1,000 resident-days (moderate) Probably no change in hospital admissions (moderate) Probably no change in mortality (moderate) Appropriateness of prescribing change uncertain (very low) Infection rates change uncertain (very low)
**Aliyu 2022** ^14^	SLR/MA	70% US	10/264	Majority UTI followed by other infection type or not reported	Education or prospective audit and feedback	Inappropriate antibiotic prescribing decrease by 13.8%, highest among UTI studies. Education and ASP algorithms for UTI indicated as most successful interventions.
**Tandan** ^15^ **2022**	SLR/MA	44% US	16/216	NR	Education, decision aids, prospective audit and feedback	31% decrease in prescribing 36% decrease in urine culture ordering Positive impact on overall and fluoroquinolone-related AMR
**Overall synthesis**	SLRs and SLRs with meta-analysis	Predominantly United States (≈40–71% of included studies), with remaining studies from Europe and other high-income settings	Each review included 5–16 studies and approximately 132–270 nursing homes	Older adult residents in nursing homes/LTCFs, with urinary tract infection (UTI) and mixed-infection cohorts most frequently studied and comparatively fewer data focused solely on respiratory or other syndromes	Multimodal antimicrobial stewardship strategies were most commonly evaluated, typically combining education, guideline dissemination, UTI-specific algorithms or decision aids, and prospective audit and feedback delivered by clinical experts	Across systematic reviews and meta-analyses, total antibiotic use generally decreased by roughly 10–30%, with improvements in guideline-concordant prescribing most evident for UTI management and reductions in urine culture ordering in studies incorporating diagnostic stewardship; effects on hospitalization, mortality, and Clostridioides difficile infection were minimal or inconsistent across reviews.

* NHs may overlap across studies; included for context, not weight.;

** GRADE certainty of evidence ratings for outcomes in parentheses; AMR, antimicrobial resistance; ASB, asymptomatic bacteriuria; DDD, defined daily dose; NHAP, nursing home associated pneumonia; NR, not reported; Rx, prescriptions; US, United States; UTI, urinary tract infection.

Consistently, urinary tract infections (UTI) interventions were the most common syndrome targeted, though categories of “all infection types” and respiratory interventions were frequent as well. These interventions moved beyond general education, specifically targeting the differentiation of asymptomatic bacteriuria from active infection to reduce unnecessary initiation, optimizing agent selection, and reducing duration of therapy. Most studies involved multimodal interventions with activities including educational strategies, clinical practice guidelines, local consensus process, audit and feedback, patient-mediated interventions, tailored interventions, continuous quality improvement, care pathways, infectious diseases team, and information/communication technology.^15^ Where these interventions were quantified by type, total interventions per study ranged from 2 to 7 with a median of 3.^15^

While these SLRs confirm the efficacy of multimodal strategies, they often lack granular guidance on which specific interventions yield the highest return on investment for resource-constrained facilities. Similar resources for other general public health initiatives are routinely compiled by the CDC.^18^ Consequently, recent research has pivoted from general efficacy reviews to expert consensus and behavioral science frameworks to prioritize implementation. Unlike the CDC Core Elements, which provide the structural framework for what a program should contain, these data offer guidance on how to prioritize actions when resources are limited.

Kruger et al conducted a multiphase modified Delphi method using a 16-member multidisciplinary panel of experts and resident representatives to facilitate the selection and adoption of interventions through consensus prioritization.^19^ Their results reflect a descending prioritization of (1) guidelines for empiric prescribing, (2) audit and feedback, (3) communication tools, (4) short-course antibiotic therapy, (5) scheduled antibiotic reassessment, and (6) clinical decision support systems. Interestingly, many interventions were not supported, including nursing home antibiograms, educational interventions, formulary review, and automatic substitution.

Enriching the perspective offered by the modified Delphi study, Crayton et al performed a systematic review to identify interventions targeting antibiotic use in LTCFs and then apply behavioral science frameworks for classifying intervention types and component behavior change techniques used.^20^ Interventions were classified as “very promising” if all outcomes were statistically significant, “quite promising” if some were significant, and “not promising” if all were non-significant. Among 20 studies, they found most promising interventions included “persuasion” (communication to stimulate action; eg., infectious diseases physician communication to reinforce messaging from education, guidelines), “enablement” (increasing capability; eg., feedback case sessions), and “education” (increasing knowledge and understanding; eg., guidelines). Additionally, they identified the most promising behavior change techniques included “feedback on behavior” (monitoring and giving information on performance behavior; eg., qualitative factors influencing prescribing) and restructuring the social environment (eg., staff role changes). The results of this SLR can facilitate planning of future interventions or refinement of current activities. Similarly, Colin et al performed a systematic review and mixed methods appraisal of implementation strategies for ASPs in LTCFs.^21^ Using data from 48 studies, they mapped strategies and outcomes to the Expert Recommendations for Implementing Change (ERIC) framework. Forty percent of studies included one of three ERIC domains including education and training, evaluative and iterative strategies, and support clinicians. Implementation frameworks, models, or theories were only used in 17% of studies. More research on dissemination and implementation strategies to localize interventions for ASPs in LTCFs are needed.

## Resources and tools for antimicrobial stewardship in LTCF

While evidence and guidance on prioritization of interventions and approaches for implementation strategy are still evolving, many tools exist to facilitate establishing ASPs and improving antimicrobial use in LTCFs (Table 2). A primary starting tool is the CDC’s Stewardship for Nursing Homes.^21^ Designed to guide the initiation and expansion of ASPs, this framework outlines seven structural components. To ensure structural support, facilities must establish Leadership Commitment (demonstrating administrative support), Accountability (identifying medical, nursing, and pharmacy leads), and Drug Expertise (accessing consultant pharmacists with infectious diseases training). Operational success relies on Action (implementing policies to improve use), Tracking (monitoring process and outcome measures), and Reporting (providing regular feedback to staff). Finally, Education ensures that clinicians, residents, and families understand the risks of resistance. Rather than a rigid checklist, the framework encourages stepwise implementation. Promisingly, NHSN Long-Term Care Facility Component annual surveys have reflected by 2018, 83% of nursing homes reported implementation of all 7 core elements of CDC core elements of antimicrobial stewardship in nursing homes.^7^ Notably, a similar outline of guidance from the Dutch Working Party on Antibiotic Policy (Dutch acronym “SWAB”) has been developed from their efforts on implementing ASPs in one of the largest nursing home care providers in the Netherlands.^22^

**Table 2.** Resources and tools for AMS in LTCF

Resource	Tool	Characteristics
CDC^19^	Core Elements of Antibiotic Stewardship for Nursing Homes	Initiation or expansion elements for ASPs in LTCFs Guidance on implementing each element
AHRQ^22^	Nursing Home Antimicrobial Stewardship Guide	Step-by-step instructions and turnkey materials Toolkits to implement, monitor, and sustain an ASP Toolkits to determine whether it is necessary to treat a potential infection with antibiotics Toolkits to help prescribing clinicians choose the right antibiotic for treating an infection Toolkit to educate and engage residents and family members
Belan et al. 2020^23^	Systematic review and inventory of tools	156 tools available free on web, focus mostly on education, patient assessment, and outcome measure

For facilities seeking ready-to-implement interventions to operationalize these elements, the Agency for Healthcare Research and Quality (AHRQ) Nursing Home Antimicrobial Stewardship Guide serves as the primary practical resource.^23^ Based on low, medium, or high availability of resources, ASPs can select among potential goal areas in identifying potential problems, helping clinicians identify an infection, helping clinicians choose the right antibiotic, and educating residents and family members. Each goal area has specific ready-to-use resources for implementation such as a “Be Smart About Antibiotics” education sheet for residents to inform them on appropriate and inappropriate use of antibiotics, risks of antibiotics, and what they can do to help be good stewards.^24^ Notably, a study by the AHRQ using these tools along with education through webinars among 439 LTCFs, reflected an overall decrease in total antibiotic courses with fluoroquinolones reflecting the largest decrease, and antibiotic course reductions were greatest at facilities with the most webinar attendance while low engagement facilities lacked an impact on course reductions.^25^ Finally, the tools and program were associated with a significant decrease in urine cultures ordered.

While the AHRQ guide provides a curated set of standard interventions, facilities with unique needs or those seeking to refine specific program aspects may require a broader repository of tools. In 2020, Belan et al published a systematic review and inventory of tools on ASPs in nursing homes.^26^ In total, 156 freely available tools were identified from the literature found in their review. The majority of tools focused on education and practical training (*n* = 58) or other actions aiming at responsible antimicrobial use (*n* = 58; diagnosis/algorithms, communication / assessment, microbiology testing, and antibiograms). Other identified tool categories included management leadership, accountability and responsibility, available expertise on infection management, monitoring and surveillance, reporting and feedback, and general recommendations for implementation. The inventory of these tools is available in their supplementary materials of their article with institution, description, format of material, and weblink provided.

Finally, regarding tools for monitoring and surveillance, recent efforts have developed LTCF-specific methods to address resource limitations. Research from Song et al used pharmacy invoices from the dispensing pharmacy for the facilities as a data source for tracking and reporting antibiotic use in nursing homes.^27^ They outline the steps of data cleaning, calculating census of bed days of care as well as using publicly available bed allocations per nursing home, and generating several antibiotic use metrics including days of antibiotic therapy, length of antibiotic therapy, rate of antibiotic starts, and antibiotic spectrum index. Finally, they created an easily accessible template for summarizing data within and across facilities. In the absence of such data, other research has reflected performing a point prevalence survey across 18 facilities required less than 2 hours for a 100-bed facility, an approach that could be repeated multiple times a year to facilitate tracking.^28^ Benchmarking antibiotic use metrics in LTCFs is still being evaluated by the CDC.^29,30^

## Conclusions

In conclusion, antimicrobial stewardship in LTCFs has rapidly evolved in recent years from establishing feasibility to optimizing impact. Evidence-based interventions, such as educational strategies, clinical practice guidelines, and audit and feedback, have demonstrated efficacy in reducing inappropriate antibiotic use and enhancing guideline adherence, especially in the management of UTIs. The availability of comprehensive tools and resources, like the CDC’s Core Elements of Antibiotic Stewardship for Nursing Homes and the AHRQ’s Nursing Home Antimicrobial Stewardship Guide, provides essential support for implementing and sustaining ASPs (ASPs). However, a critical gap remains between identifying effective interventions and successfully implementing them. As noted in our review of implementation strategies, only 17% of studies utilized formal frameworks to guide adoption, and research on behavioral drivers (eg., “persuasion,” “enablement”) is limited compared to traditional clinical outcomes. Consequently, future research must pivot from testing new clinical guidelines to evaluating the implementation strategies required to change prescribing culture. Specifically, rigorous investigation is needed to determine which behavioral techniques most effectively sustain stewardship in settings with high staff turnover and limited infectious diseases expertise.
